# New Materials and Technologies for Durability and Conservation of Building Heritage

**DOI:** 10.3390/ma16031190

**Published:** 2023-01-30

**Authors:** Luigi Coppola, Tiziano Bellezze, Alberto Belli, Alessandra Bianco, Elisa Blasi, Miriam Cappello, Domenico Caputo, Mehdi Chougan, Denny Coffetti, Bartolomeo Coppola, Valeria Corinaldesi, Alberto D’Amore, Valeria Daniele, Luciano Di Maio, Luca Di Palma, Jacopo Donnini, Giuseppe Ferrara, Sara Filippi, Matteo Gastaldi, Nicola Generosi, Chiara Giosuè, Loredana Incarnato, Francesca Lamastra, Barbara Liguori, Ludovico Macera, Qaisar Maqbool, Maria Cristina Mascolo, Letterio Mavilia, Alida Mazzoli, Franco Medici, Alessandra Mobili, Giampiero Montesperelli, Giorgio Pia, Elena Redaelli, Maria Letizia Ruello, Paola Scarfato, Giuliana Taglieri, Francesca Tittarelli, Jean-Marc Tulliani, Antonino Valenza

**Affiliations:** 1 Department of Engineering and Applied Sciences, University of Bergamo, INSTM R.U., 24044 Dalmine, Italy; 2 Department of Materials, Environmental Sciences and Urban Planning, Università Politecnica delle Marche, INSTM R.U., 60131 Ancona, Italy; 3 Lince Laboratory, Department of Applied Science and Technology, Politecnico di Torino, INSTM R.U., 10129 Turin, Italy; 4 Department of Enterprise Engineering “Mario Lucertini”, University of Roma “Tor Vergata”, INSTM R.U., 00133 Rome, Italy; 5 Department of Civil and Industrial Engineering, University of Pisa, 56122 Pisa, Italy; 6 Department of Chemical, Materials and Industrial Engineering, University of Naples Federico II, 80125 Napoli, Italy; 7 Department of Engineering, University of Campania “Luigi Vanvitelli”, 81031 Aversa, Italy; 8 Department of Industrial and Information Engineering and Economics, University of L’Aquila, 67100 L’Aquila, Italy; 9 Department of Industrial Engineering, University of Salerno, 84084 Fisciano, Italy; 10 Department of Chemical Engineering Materials & Environment, Sapienza University of Rome, 00184 Rome, Italy; 11 Department of Chemistry, Materials and Chemical Engineering “G. Natta”, Politecnico di Milano, 20133 Milano, Italy; 12 Department of Civil and Mechanical Engineering, University of Cassino and Lazio Meridionale, 03043 Cassino, Italy; 13 Department of Heritage-Architecture-Urbanism, University of Reggio Calabria “Mediterranea”, 89124 Reggio Calabria, Italy; 14 Department of Mechanical, Chemical and Materials Engineering, University of Cagliari, 09123 Cagliari, Italy; 15 Department of Engineering, University of Palermo, 90123 Palermo, Italy

**Keywords:** durability, concrete structures, sustainability, new materials

## Abstract

The increase in concrete structures’ durability is a milestone to improve the sustainability of buildings and infrastructures. In order to ensure a prolonged service life, it is necessary to detect the deterioration of materials by means of monitoring systems aimed at evaluating not only the penetration of aggressive substances into concrete but also the corrosion of carbon-steel reinforcement. Therefore, proper data collection makes it possible to plan suitable restoration works which can be carried out with traditional or innovative techniques and materials. This work focuses on building heritage and it highlights the most recent findings for the conservation and restoration of reinforced concrete structures and masonry buildings.

## 1. Introduction

The construction sector depletes 40% of the planet’s energy resources [1,2]. To decrease the energy consumption of buildings in order to improve their sustainability, a key aspect to consider is the durability of concrete structures (such as buildings, tunnels and bridges), since it highly affects their service life [3].

The durability of concrete is strongly influenced by the interactions between concrete itself and the surrounding environment, which is rich in contaminants [4]. Among several causes, the most common are water penetration, since water represents the main carrier of aggressive agents [5,6,7]; CO_2_ penetration, which determines cement matrix carbonation and, in presence of moist air, is responsible for reinforcement corrosion [8]; chloride penetration, which initiates reinforcement corrosion [9]; heavy metal contact and incorporation; and fire development, which degrades the concrete itself [10].

**Figure 1:**
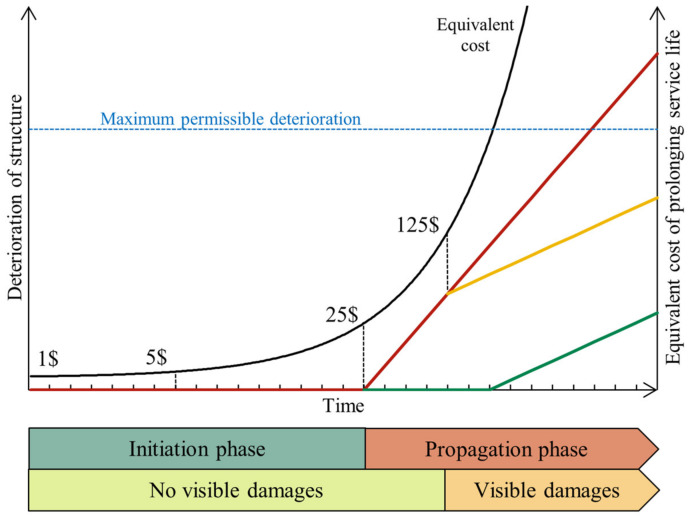
De Sitter’s “Law of Fives” in Tuutti’s diagram. Re-elaborated from [11,13].

From “The Law of Fives” by De Sitter (Figure 1), it is well known that the costs of repairing a concrete structure grow exponentially if the works are done long after the first damage appears, especially in the case of reinforced-concrete-based monuments [11]. Therefore, it becomes clear that monitoring the penetration of aggressive agents in concrete is important to accelerate interventions, which can in turn reduce maintenance costs and increase the structural safety of structures [12]. Additionally, the Italian Technical Regulations for Buildings (approved on 17 January 2018) reports in its Chapter 2 “Safety and Expected Performance” that an adequate level of durability can be guaranteed by adopting passive and active control systems.

The purpose of this review is to analyze the latest findings on materials and techniques devoted to ensuring prolonged durability and proper conservation of reinforced-concrete-based heritage.

## 2. Monitoring Systems for Durable Concrete Structures

The diagnosis of durability should take place through a methodological path primarily based on the visual analysis of the structure to detect possible visible degradation phenomena and on the acquisition of historical–geographic data. These data are complementary to the results obtained by in situ and laboratory tests to assess the diagnosis and to define the maintenance interventions to be carried out.

On one hand, the main destructive tests performed on samples extracted from the structure and analyzed in laboratory include the chemical (chromatography, X-ray diffraction, thermal analysis, FT-IR spectroscopy), physical (mercury intrusion porosimetry), morphological (optical and electronic microscopy) and elasto-mechanical (modulus of elasticity, compressive and tensile strength, etc.) characterization of materials.

On the other hand, on-site non-destructive tests, mainly focused on elastic–mechanical characteristics of the concrete, are endoscopy, sclerometry, thermography, pull out, and sonic and ultrasonic investigations. Colorimetric methodologies [14] are used to detect the penetration depth of CO_2_ and chlorides, whereas potential mapping and resistivity measurements are used to monitor reinforcement corrosion.

However, continuously monitoring selected parameters through embedded probes, in order to detect the initiation of degradation as early as possible, can further decrease costs and improve the safety of structures, because it increases the chance of detecting any significant variation of the chosen parameter related to a degradation process. Therefore, numerous probes to be embedded in concrete have been developed, such as electrical resistance probes (ERP) [15], macro-couple probes, pseudo reference electrodes to measure free corrosion potential (activated Ti mixed metal oxide (MMO), Zn) and multi-parametric sensors [16].

**Figure 2:**
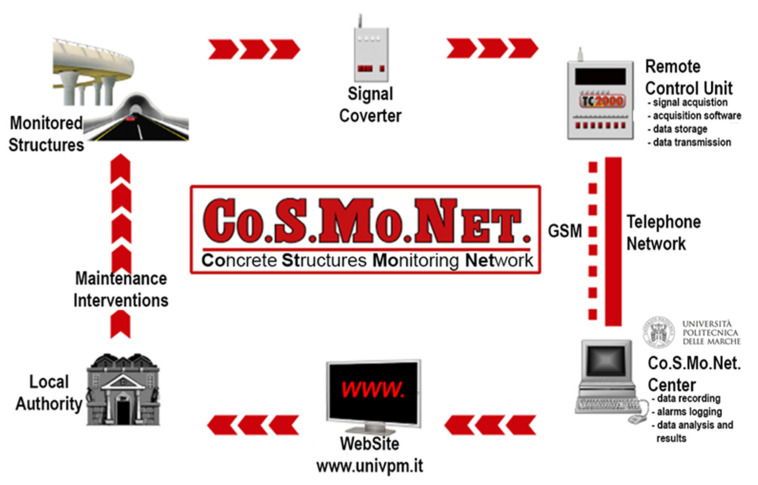
The Co.S.Mo.Net monitoring system.

In particular, the free corrosion potential is easy to measure and can be used to control the durability of reinforced concrete structures, since it is sensitive not only to the corrosive state of the reinforcements but also to the water saturation degree of the concrete between the monitored reinforcement and the embedded probe. It is worthy to underline that water is the main factor influencing the durability of structures, since it transports the main aggressive agents and it is the medium in which deterioration reactions take place. A continuous monitoring system for preventive and planned maintenance of structures based on simple measurement of the free corrosion potential has been already proposed (i.e., CoSMoNet [17], Figure 2) and successfully applied to several concrete structures. The system provides signals, revealed by embedded electrodes and sent to a peripheral device for remote reading, where data are suitably analyzed and stored and finally sent to a monitoring station for their processing. Special alerts are arranged when the variation in the measured values exceeds a maximum threshold previously established. In this way, a continuous monitoring of different reinforced concrete structures located everywhere in the world can be carried out from the same monitoring station.

The most recently proposed advanced non-destructive testing tools and procedures for structural health monitoring (SHM) are sensorized non-metallic reinforcement systems for concrete [18], computer vision [19]**,** also combined with thermography [20,21], and electrical resistivity [22] (i.e., EU Project: EnDurCrete [23]). Electrical resistivity, which can be determined by electrochemical impedance spectroscopy (EIS), is getting much attention compared with other methods. Indeed, the EIS characteristics make it possible to exploit the self-sensing capability of concrete, which means its ability to sense its own condition (e.g., cracks, water penetration, strain) [24]. This ability can be enhanced by increasing the electrical conductivity of concrete by adding conductive materials such as fillers/fibers [25,26,27]; higher concrete conductivity allows the monitoring of concrete structures by means of low-cost instrumentation given the higher signal-to-noise ratio (SNR) that can be obtained [28]. Concerning fillers, carbon nanotubes [29], graphene [30], carbon black [31,32], and nickel powder [33] have been proven to increase the electrical conductivity of mortars/concretes besides their mechanical performance and durability [34,35,36]. Concerning fibers, Xie et al. [37] focused on the relationship between fiber content and electrical conductivity of the cement-based composite, highlighting a remarkable reduction in the electrical resistivity if the fiber content is higher than a threshold due to the increase in electrical contact paths between the fibers. Comparing steel and carbon fibers [38], a recently published work has shown the best effectiveness is in recycled carbon fibers (RCF) compared with virgin carbon fibers (VCF) and brass-coated fibers (BSF) in noticeably reducing concrete resistivity even at low fiber dosages [39]. This result can be ascribed to the high number of carbon micro-particles on the fibers’ surface, which increase the specific conductive surface of RCF [40].

## 3. Sensors to Evaluate the Carbonation of Concrete

Carbonation is a two-step reaction process between: (i) atmospheric CO_2_ (about 419 ppm by volume in December 2022, measured at the Mauna Loa Observatory in Hawaii, USA [41]) with water present in concrete capillary pores which leads to the formation of carbonic acid (H_2_CO_3_) and (ii), by reaction of H_2_CO_3_ with calcium compounds (primarily, portlandite (Ca(OH)_2_)), to produce calcium carbonate (CaCO_3_) and water. The decrease in the portlandite concentration reduces the pH of the pore solution to a value of about 8.3 after complete carbonation of concrete [42]. However, reinforced concrete structures require a high pH to maintain the stability of the passivated layer on top of the rebars. When the pH value decreases, this protective oxide layer breaks and corrosion can occur. In addition, the corrosion is accelerated if the steel rebars are exposed to aggressive agents such as chloride ions. The corrosion produces expansive iron oxides/hydroxides and causes, in hardened concrete, internal tensile stresses, cracking and spalling of the concrete cover. Exposure conditions, in particular relative humidity (RH), play a fundamental role in the carbonation depth and the amount of CO_2_ reacted over time, as carbonation is favored in the RH value range from 40 to 90% [42]. The carbonation rate is also influenced by the mix design of concrete (type and dosage of cement, water-to-cement ratio and, thus, porosity of concrete), as well as curing conditions. In most structures made using good-quality concrete, carbonation needs many years to extend as far as the depth of the rebars [43].

The most common method for the evaluation of carbonation is destructive and involves a 1% (*w*/*v*) solution of phenolphthalein in ethanol, usually sprayed on cylindrical samples cored from concrete structures [44]. This pH indicator varies its color from pink/purple (in a non-carbonated environment) to colorless when the pH value is below 9.0–9.5, evidencing the carbonated area. Sensors working in accordance with electrochemical principles, like potential, polarization resistance and electrochemical impedance measurements [45], fiber-optic sensors based on Fabry–Perot interferometer strain sensors [46] or acoustic emission [47] are also used to assess the corrosion rate of rebars in reinforced concrete structures. However, these sensors are placed in direct contact with steel reinforcements and can alert only after the initiation of the corrosion process, which is in most cases already too late. Thus, preventing rebar damage and early detection of the carbonation depth of a concrete cover before rebars are reached is of paramount importance. A state-of-the-art pH determination in hardened concrete was published by Behnood et al. [48]**,** where destructive (based on pore solution extraction/leaching) and non-destructive (with embedded sensors) approaches were deeply illustrated.

Even if potentiometric sensors for pH measurement are commonly used in many areas, their use to monitor the pH of concrete pore solution is somewhat limited [49]. Iridium/iridium oxide (IrO_x_) electrodes [50,51,52,53] and screen-printed Ag/Ag_2_O [53] or IrO_x_ [54] sensors were studied with this aim.

Electrode pH probes to monitor pH evolution at different depths were also investigated. For example, a fiber-optic sensor based on a pH-sensitive layer entrapping a pH indicator which changed color in response to the carbonation state of the cementitious matrix was described by Habel and Krebber [55]. Other papers also illustrated the interest in using sol-gel optic fiber sensors for pH monitoring in cementitious materials [56,57,58]. McPolin et al. produced by a sol-gel method an optic fiber probe with a cresol-red indicator dye trapped inside (pH variation between 8 and 13), embedded it in cement mortar samples, and monitored pH changes over 18 months [59]. Khalil et al. presented a pH sensor based on meso-tetraarylporpholactone, which shifted color in the 11.5–13.2 pH range [60]. Srinivasan et al. used a sol-gel/TNBS (trinitrobenzenesulfonic acid) composite that showed a variation in color in the pH range from 12 to 14 [61]. Finally, Inserra et al. [62] developed an optical pH probe with a pH-sensitive dye embedded in a silica monolith made by a sol-gel method. Alizarin yellow, which changes color from yellow to red when the pH ranges from 10 to 12, was used for this purpose.

## 4. Electrochemical Techniques for Repairing Reinforced Concrete Structures

In some cases, specifically when concrete damage is due to reinforcement corrosion, conventional repair techniques may not be enough to guarantee the required service life of the intervention. For instance, this may be related to the inadequacy of concrete cover thickness, high aggressiveness of the environment, or the need to remove large quantities of structurally sound concrete. In such cases, electrochemical techniques can be an advantageous option, since they can interrupt corrosion propagation, leaving the concrete in place, provided it has not been damaged yet.

Electrochemical techniques can be permanent or temporary [63,64,65,66]. The main permanent technique is cathodic protection, which is based on the application of a small current density (up to a few tens of mA/m^2^) to protect steel reinforcement. Re-alkalisation and chloride removal are temporary treatments that rely on the application of a much higher current density (up to few A/m^2^) for several weeks or a few months to change concrete’s composition and restore its ability to protect the reinforcement.

All techniques rely on the application of a cathodic current to the reinforcement, usually supplied by a metallic anode placed on the concrete surface (for cathodic protection, the anode is embedded in a layer of mortar, while for temporary techniques the anode is embedded in cellulose pulp and removed at the end of the treatment). The main effect of the current is a reduction in steel potential, and a subsequent reduction in the corrosion rate. This effect is temporary (it is lost if the current is switched off); as long as the current is circulating, the rate of the anodic reaction (oxidation of iron) is depressed and the rate of the cathodic reaction (production of OH-) is increased. If very negative potential values are reached, the cathodic reaction of hydrogen evolution can also occur, and its consequences should be carefully evaluated in the case of high-strength steel bars in prestressed concrete structures [67,68].

The applied current also changes the properties of the concrete. The already mentioned production of alkalinity increases the pH at the steel–concrete interface, strengthening the protection so that short interruptions of the applied current can be tolerated. Moreover, the movement of anions in opposite directions to the current spreads alkalinity over larger areas of concrete and takes chlorides away from the rebar. These effects are beneficial, but marginal, in the case of cathodic protection; conversely, they are of primary importance for temporary treatments.

All electrochemical techniques need monitoring to check that the current is flowing and it is enough to protect the reinforcement (this also applies to re-alkalisation and chloride removal, where, in addition, monitoring of the corrosion conditions of the rebar after the treatment is advisable) [69].

Traditionally, cathodic protection is applied to structures suffering chloride-induced corrosion, typically slabs of bridges and viaducts or marine structures; however, it can be conveniently applied to carbonated concrete as well. The use of sacrificial anodes is also possible, by inserting discrete elements of zinc into the concrete and connecting them to the rebars (in this case, the duration is limited by the consumption of the anode material) [70,71,72].

Temporary treatments are used when it is not possible to apply a permanent anode, either for technical or aesthetical requirements. They can be used on complex shapes (e.g., statues) or if the texture of the surface cannot be changed (e.g., fair-faced concrete). Their effectiveness is more limited in time, and also because the cause of corrosion (e.g., carbonation or chloride penetration) continues after the treatment; it is also reduced if corrosion products are abundant at the steel–concrete interface [73,74,75].

## 5. Multi-Functional Graphene-Based Cement Composites for Durable Repaired Structures

In the last decade, graphene-based cement composites (GBCCs) have been strongly investigated due to their enhanced mechanical properties, often combined with smart properties (such as electrical, thermal, piezoresistive and electromagnetic properties), fire resistance and freeze-thaw resistance [76]. Moreover, very recently, it has also been proved that GBCCs show reduced permeability and chloride penetration. Several novel applications are expected in the near future for graphene-based cement nanocomposites as smart repair materials in the fields of offshore structures, geothermal piles, radiant systems, smart pavements, deicing roads and electromagnetic shielding [77,78,79].

The family of graphene-based materials (GBMs) is wide and includes several nanomaterials, classified on the basis of three key parameters: number of graphene layers, average lateral size and C/O atomic ratio [80].

A number of different characterization techniques are thus needed to fully characterize GBMs, such as Raman spectroscopy, infrared (IR) spectroscopy, X-ray photoelectron spectroscopy (XPS), and X-ray diffraction (XRD) combined with an extensive investigation by means of scanning electron microscopy (SEM), transmission electron microscopy (TEM) and atomic force microscopy (AFM) [81]. Recently, inelastic neutron scattering has been used to assess the hydration of GBCCS and the interactions between fillers, additives and hydrated cement phases [82].

One of the main issues with graphene-based materials is the workability of the fresh mortars, which is severely dependent on both the type and amount of the loaded GBMs. Usually, due to the tendency of these nanofillers to absorb water molecules, the flowability of GBM-modified cementitious admixtures is reduced; the extent depends on several different parameters, but mostly on the C/O ratio [83]. Furthermore, a good interaction between carbonaceous filler and matrix is mandatory to enhance the mechanical and functional properties of the composites. In fact, these fillers have often a strong tendency to agglomerate under attractive forces (e.g., Van der Waals), hindering the positive effect of the filler. The use of a natural rubber latex aqueous dispersion has been exploited to avoid the confinement of multiwall carbon nanotubes (MWCNTs) and reduced graphene oxide (rGO) in the production of multifunctional cement mortars with enhanced piezoresistive properties [82]. This new approach makes possible either avoiding filler aggregation or obtaining a three-dimensional network with fillers located on the single latex particles in a continuous rubbery phase.

Analogously, scientists involved in this adventurous field are aware that the mechanical and physical properties of hardened graphene-based cement nanocomposites strictly depend on the selection of type (i.e., size, composition, thickness, roughness, dispersibility, …) and dosage (usually between 0.01% and 1% by weight of cement) of the loaded graphene-based materials [84]. For this reason, although in the literature the great potential of these graphene-based cement composites has been fully assessed, the results in terms of final mechanical and physical properties are still unpredictable. Presently, most studies on GBCCs have been focused on cements and mortars. For example, cementitious composites filled with multi-layer graphenes (MLGs) showed a 54% increase in compressive strength and a 21% reinforcement in flexural strength, mainly attributed to extensive and strong bonding between the nanofiller and the matrix combined with the lowered orientation index of portlandite crystals [85]. Moreover, GBCCs also demonstrated a reduced chloride ion diffusion, associated with improved hydrophobicity, accelerated hydration kinetics and enhanced densification of the manufacts [26]. Recently, several papers have focused on the effect of GBMs in mortars or concrete on the diffusion of Cl^−^ ions in cementitious matrix [86,87]. Du and Pang [88] showed that the addition of 1.5% by weight of cement of such nanofillers in concrete promotes a decrease in chloride depth ranging from 60 to 70% relative to plain concrete. Similarly, Dimov et al. [89] published an outstanding, extensive paper on graphene-modified concrete. The authors clearly demonstrated an increase of up to 146% in the compressive and 79.5% in the flexural strength, combined with enhanced electrical and thermal conductivity; moreover, the graphene-modified concrete showed nearly 400% decreased water permeability. These results definitely open up the emerging field of multifunctional nanoengineered concrete for a more sustainable construction industry.

Obviously, safety, costs and life cycle assessment (LCA) should be rigorously evaluated for large-scale applications [90,91].

## 6. Inorganic Matrix Composite Systems for the Repair and Strengthening of Existing Structures

**Figure 3:**
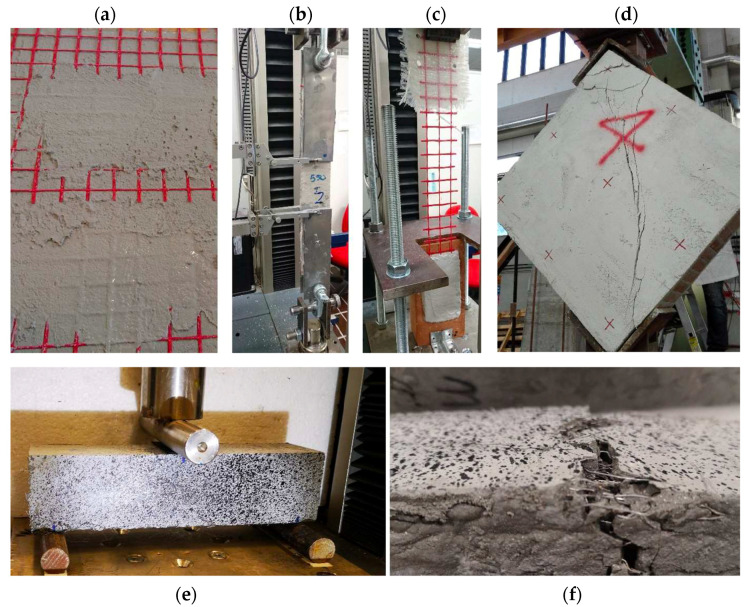
Inorganic matrix composite systems: (**a**) FRCM made of glass fabric and cementitious mortar; (**b**,**c**) mechanical characterization tests of FRCM systems; (**d**) diagonal compression test on masonry reinforced with CRM; (**e**,**f**) three-point bending test on a UHPFRC specimen and detail of the steel fibers after cracking.

The development of new inorganic matrix composite systems with strong mechanical performance has introduced into the construction industry new potential tools to repair and improve both the resistance and durability of existing concrete and masonry structures [92,93,94]. The advantages of using these systems, relative to more traditional ones, lie in their high strength/weight ratio, ease of application, greater compatibility with the substrate and better resistance to high temperatures and fire exposure. Within this new class of inorganic-based materials, those that have been developed and spread more in the construction sector are FRCM (fabric-reinforced cementitious matrix), CRM (composite reinforced mortar) and UHPFRC (ultra-high-performance fiber-reinforced concrete), Figure 3.

FRCM systems consist of an inorganic matrix (usually cement or lime-based mortar) reinforced with fabrics in the form of open grids (made of carbon, glass, basalt or PBO fibers). These systems, which have a thickness of between 10 and 15 mm, are specifically designed to be applied as external reinforcements on existing masonry or concrete structures (Figure 3a–c). As compared with more traditional strengthening solutions, such as steel-reinforced concrete plasters, FRCMs are more durable, since they have no problems related to the corrosion of the internal reinforcement and they offer better efficiency in terms of resistance/weight, as well as reversibility of the intervention and ease of application.

In recent years, the scientific community has paid great attention to this new class of composite materials, and some guidelines giving instructions on how to design a strengthening intervention with FRCM have been provided by the American Concrete Institute (ACI 549.4R-13) and Italian CNR (DT215/2018) [95,96], while acceptance criteria and indications of characterization methods are given by the ICC Evaluation Service (AC434.13), the Italian CSLLPP, and Rilem TC 250 [97,98,99]. Several studies have focused on the mechanical characterization of FRCM [100], on the bond at the interface between the internal reinforcement and the inorganic matrix [101,102,103] and on the effectiveness of different FRCM systems to repair and strengthen masonry or concrete elements [104,105,106,107,108]. There are still other issues to be further investigated, such as the long-term behavior and durability of FRCM systems when exposed to aggressive environments [109,110,111,112,113], high temperature or fire scenarios [114,115].

CRM systems are made of a pre-impregnated composite mesh (FRP) embedded within inorganic mortar (usually lime-based) with a compressive strength between 5 and 20 MPa. In these systems, the FRP mesh bears the tensile stresses, while the structural mortar is responsible for the stress transfer between the composite grid and the substrate. The transfer of stresses between the support to be reinforced and the reinforcement mesh is also guaranteed by the presence of connectors, which ensure the structural collaboration between the wall element and the reinforced plaster. The total thickness of CRM systems is usually between 30 mm and 50 mm. The most-used fibers are made of AR glass, carbon or basalt, coated with a thermosetting polymer matrix. These systems have had strong development in the last 5 years and a high diffusion especially into the Italian construction market. Further spread of these systems has also been facilitated by the recent technical guidelines for identification, qualification and acceptance control of CRM issued by the Italian CSLLPP [116].

Although recently introduced, CRM showed promising results in improving the mechanical performance of different masonry structures, such as walls and arches [117,118,119]. Reinforcement can be applied on one side only or on both sides of the masonry (Figure 3d). Diagonal compression tests on masonry walls showed an increase of the shear capacity from 42% to 85% for a single-sided configuration and from 138% up to 288% in a double-sided configuration, relative to unreinforced masonry panels [120].

UHPFRCs are advanced cementitious materials with excellent mechanical properties: they can achieve compressive strength greater than 150 MPa, flexural strength higher than 40 MPa and considerable tensile-strain-hardening behavior [121,122]. Due to their superior properties, UHPFRCs are being increasingly used to produce thin and extremely resistant structural components [123,124] or as overlay systems to repair existing concrete elements such as pillars, beams and slabs (Figure 3e,f) and to improve their mechanical performance and durability properties [125,126,127,128]. UHPFRC mixtures are characterized by a significant amount of cement (>600 kg/m^3^), small size aggregates (<6 mm), binders (fly ash, silica fume, reactive powder), superplasticizers and a low water/cement ratio (w/c < 0.2). Steel fibers (length of 10÷20 mm) are usually added with a dosage of about 2–3% by volume to significantly improve tensile strength and strain capacity [129,130,131,132,133]. A well-designed UHPFRC is able to exhibit, when subjected to uniaxial tensile strength, a strain-hardening behavior in the post-cracking phase. This phenomenon, observed by Wille at al. and Naaman and Reinhardt [121,122], is generally accompanied by the formation of multiple transversal cracks at different specimen cross-sections. For this reason, the tensile strength of these materials cannot be neglected during the design phase (as is the case of ordinary concrete). Some recommendations on how to perform direct tensile tests on UHPFRC materials have been provided by a few national guidelines, such as the French AFGC-SETRA [134] and the Japanese JSCE [135]. However, the complete mechanical characterization of UHPFRC (especially regarding its tensile strength) is still an open issue.

## 7. Fibrous and Particle Systems for Repairing Mortars

**Figure 4:**
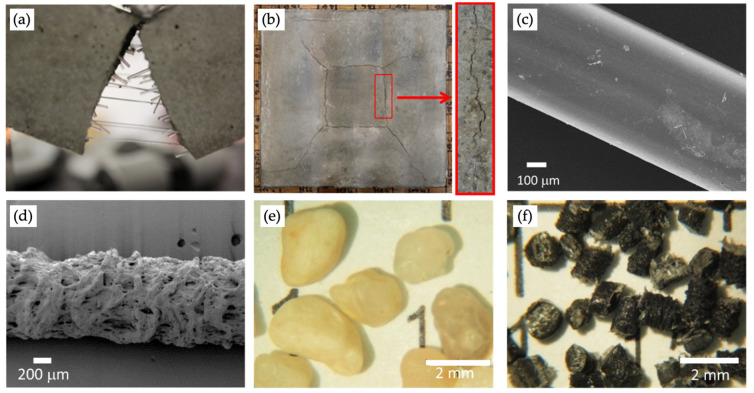
(**a**) Polymeric fibers bridging the two sides of a mortar sample after a flexural test; (**b**) shrinkage cracking phenomena occurring after an accelerated test; (**c**) FE-SEM micrograph of a smooth PP fiber; (**d**) FE-SEM micrograph of an engineered roughened fiber; (**e**) silica sand; and (**f**) artificial aggregates prepared using a secondary raw material.

Different strategies can be used to avoid or to reduce structure degradation or to repair structures that are already damaged [136]. The use of fibrous and particle systems are two viable strategies to overcome most of the issues of cementitious structures. In particular, steel fibers or polymeric fibers with a high elastic modulus are mainly used in concrete to increase its fracture toughness and flexural post-cracking behaviour (Figure 4a). On the other side, randomly oriented polymeric fibers having a low elastic modulus are generally used into cementitious mortars to avoid shrinkage cracking phenomena (Figure 4b) [137,138]. Considering shrinkage cracking phenomena and mechanical property improvements, fiber amounts and shape (geometry, cross-section and surface texture) are the two most important parameters that should be taken into account. Fibers can dramatically decrease crack numbers and width without excessively compromising mortar’s fresh properties. However, the interfacial transition zone (ITZ) between fibers and the cementitious matrix is crucial. To contrast cracking phenomena, polymeric fibers of different nature are generally used (in particular, PET, PA, PE and PP), with some recent innovations like nanocomposite polymeric fibers which have better mechanical properties compared with conventional polymeric fibers [139,140,141,142]. Indeed, most of the polymeric fibers are smooth and have weak adhesion with the matrix. Therefore, several strategies can be adopted to improve fiber/matrix adhesion: fiber surface roughness and geometry modification or improvement of the chemical affinity between fibers and the matrix [143,144]. Meanwhile, using natural fibers (hemp, flax, sisal, cellulose, bamboo etc.), there are more interactions between fibers and the matrix thanks to the interlocking positions offered by lumen and pores already present in natural fibers [145,146,147,148]. Moreover, material choice is also fundamental in terms of sustainability. Plastic fibers produced starting from recycled polymers should be preferred to virgin polymers, and natural fibers should be preferred to mineral fibers that are produced at high temperatures (e.g., basalt) [149,150,151,152,153,154]. Indeed, the reuse of wastes deriving from different streams (e.g., polymeric, sewage sludge, construction and demolition, etc.) is an effective strategy to improve the sustainability of the building and construction sector [155,156]. Some efforts have been made to extend the strong potential of plastic waste obtained by plasticization and densification of the polymeric fraction of municipal solid waste (MSW) in the field of lightweight concrete [157]. Satisfactory adhesion and good compatibility between plastic aggregates and a cement matrix were confirmed by SEM analysis (Figure 4). Even with a small amount of plastic substitution (about 10%), the concrete was compliant with Italian standards for structural use; a significant improvement in tensile strength can be achieved thanks to the fiber-like behavior of plastic waste [157].

A further improvement in terms of sustainability can derive from the substitution of traditional aggregates used for cementitious mortars with more innovative ones. Indeed, by varying the nature, the morphology and the chemical properties of the aggregates, several benefits can be obtained. As for fibers, aggregates can also be prepared starting from secondary raw materials [152,158,159]. Using porous and lightweight aggregates, it is possible to reduce the specific weight of the composite [158,160,161]. Moreover, porous aggregates can be saturated to be used as water reservoirs for internal curing or as carriers for self-healing agents [162,163]. Moreover, hygro-thermal properties can be improved by opportunely tuning aggregate amounts, reducing thermal conductivity and increasing water vapor permeability [159,164,165]. Finally, using plastic aggregates is also possible to improve the impact and shock resistance of cementitious mortars [166].

## 8. Nanolime-Based Formulations for the Conservation of Cultural Heritage

Silica fume (SF) and natural pozzolans (NP) have been widely used as substitutes for Portland cement (OPC) for concrete manufacturing, in relation to their advantageous properties, including reduced environmental impact, low heat of hydration, low water permeability, high chemical resistance and resistance against the alkali–silica reaction, improved fresh properties, low shrinkage and reduced cost [167,168]. In particular, in such cases, both SF and NP play a fundamental role in improving the packaging density of the solids, but their primary role is to provide additional calcium silicate hydrate (C-S-H) through reaction with water and the calcium hydroxide coming from the hydration of OPC [169,170,171].

The interaction, in terms of the production of C-S-H, between NP and SF and an aqueous suspension of nanolime particles (nCH) produced in the laboratory by means of a patented, eco-friendly and one-step synthetic route based on an ion-exchange process makes it possible to obtain large amounts of product [172,173,174,175,176]. The interaction between nCH and NP/SF is realized in water, working at room temperature, considering different nCH/NP and nCH/SF ratios, corresponding to 1:1 and 1:2 in weight, and a water/solid ratio (W/S) equal to 6. The obtained mixtures are analyzed at different aging times, from 30 min up to 120 days. Both the as-received commercial NP/SF and the synthesized nCH are characterized by means of several techniques, such as XRD, XRF and transmission electron microscopy (TEM) analyses. The produced mixtures are investigated in terms of the phase composition of the formed hydrates at different aging times. Scanning electron microscopy (SEM) is used to investigate their morphological features as well. In addition, surface measurements (BET) are performed.

**Figure 5:**
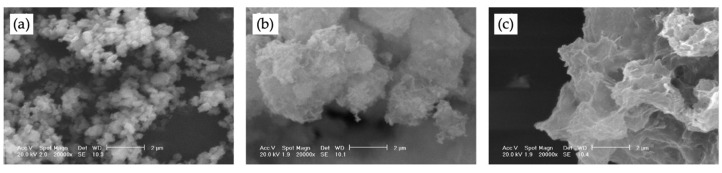
SEM images of nCH/NP mixtures at different hydration times: (**a**) after 30 min; (**b**) after 28 days, considering a nCH/NP ratio of 1:1; (**c**) after 28 days, considering a nCH/NP ratio of 1:2. Scale bar: 2 μm.

From XRF, SF is characterized by 94.82% SiO_2_, 0.79% Fe_2_O_3_ and minor amounts of MgO, K_2_O, CaO and Al_2_O_3_, while the NP is composed of 69.17% SiO_2_, 1.16% TiO_2_ and minor amounts of Al_2_O_3_, K_2_O, SO_3_ and Fe_2_O_3_. BET analyses reveal surface area values of about 12.5 m^2^/g and 60 m^2^/g for SF and NP, respectively. The synthesized nCH appears pure and crystalline, in the form of hexagonal lamellas consisting of primary nanoparticles with dimensions ≤ 10 nm. Moreover, the aqueous suspensions of nCH show a high reactivity, guaranteeing a complete conversion into calcium carbonate, in the form of pure calcite, in a few hours, even in low-relative-humidity conditions [175]. As concerns the nCH/SF mixtures, from the XRD spectra the initial formation of C-S-H is evident after only 3 and 14 days of hydration, considering nCH/SF ratios of 1:2 and 1:1, respectively. In the sample characterized by a nCH/SF ratio of 1:2, after 14 days all the Ca(OH)_2_ is consumed and the C-S-H formation proceeds in time appearing more crystalline, mainly in the C-S-H(I) form. The mixture with NP is characterized by a high reaction rate, especially at low hydration times, with the formation of C-S-H after only 1 day of aging. Nevertheless, the Ca(OH)_2_ is not completely consumed up to 14 and 60 days, considering nCH/NP ratios of 1:2 and 1:1, respectively. The obtained results are very promising if compared with the literature data, which indicate a progressive reduction in free lime over time, leading to a total consumption at 90 and 365 days when silica fume or natural pozzolan are employed, respectively. Considering BET results, specific surface values of about 121 m^2^/g and 154 m^2^/g are obtained, referring to nCH/SF and nCH/NP mixtures with 1:2 ratios, respectively. Finally, SEM images of nCH/SF mixtures after 28 days of hydration show the formation of crumped foils, with a thickness of few nanometers, characterized by the typical fibrous structure. In the nCH/NP mixtures, the formation of highly wrinkled layers covering all particles, constituted by marked crumple and rough-edge surfaces, is observed as well (Figure 5).

## 9. Future Trends

In the development of a conservative strategy for building heritage, evaluation of the state of conservation of the materials plays a central role. This survey is done using both monitoring techniques for the existing structures before the restoration work and evaluation of the effectiveness of the maintenance already carried out in the past. With the aim of a new conservation philosophy, all techniques, traditional or innovative, for acquiring information about the building’s health conditions are fundamental, and also using strategies originally developed for other sectors (i.e., digital twins, artificial neural networks). Furthermore, the acquisition of data makes it possible to provide a large functional database for the development and refinement of the repairing materials and techniques of the future.

Therefore, starting from the knowledge of the building, the choice of traditional or innovative techniques will have to take into account, in addition to all the issues related to the quality of the restoration work, also sustainability, thus preferring the use of low-environmental-impact solutions (i.e., with low CO_2_ emissions, limited consumption of energy and natural resources, reuse of waste products and use of recyclable materials) capable of guaranteeing a prolonged service life for the building as discussed in a previous review by the authors [3].

## 10. Conclusions

This review highlights the most recent findings for the conservation and restoration of building heritage. In particular, it focuses on the need to guarantee a proper diagnosis of the durability of existing structures, and it shows that it is possible to preserve building heritage by using electrochemical techniques (when reinforced concrete elements are carbonated and/or in contact with chlorides). Moreover, some innovative techniques and materials for repairing building heritage (such as smart graphene-based materials, inorganic matrix composites, nano-limes and cementitious systems containing fibrous or particle reinforcements) are presented.

## Data Availability

Not applicable.
